# Intra-Articular Administration of Recombinant Human Proteoglycan 4 (rhPRG4) as a Potential Therapy for Temporomandibular Joint Osteoarthritis: A Preclinical Histopathological Study

**DOI:** 10.3390/ijms26199305

**Published:** 2025-09-23

**Authors:** Veronica Iturriaga, Bélgica Vásquez, Schilin Wen, Thomas Bornhardt, Javiera Navarrete, Mariano del Sol

**Affiliations:** 1 Department of Integral Adult Care Dentistry, Temporomandibular Disorder and Orofacial Pain Program, Universidad de La Frontera, Temuco 4811230, Chile; thomas.bornhardt@ufrontera.cl; 2 Sleep & Pain Research Group, Universidad de La Frontera, Temuco 4811230, Chile; 3 Center of Excellence in Morphological and Surgical Studies, Universidad de La Frontera, Temuco 4811230, Chile; mariano.delsol@ufrontera.cl; 4 Department of Basic Sciences, Faculty of Medicine, Universidad de La Frontera, Temuco 4811230, Chile; 5 Grupo de Investigación de Pregrado en Odontología, Facultad de Ciencias de la Salud (FACSA), Universidad Autónoma de Chile, Temuco 4810101, Chile; schilin.wen@cloud.uautonoma.cl; 6 Doctoral Program in Morphological Sciences, Faculty of Medicine, Universidad de La Frontera, Temuco 4811230, Chile; j.navarrete10@ufromail.cl

**Keywords:** temporomandibular joint, temporomandibular joint disorders, osteoarthritis, proteoglycan 4, lubricin, cartilage

## Abstract

The proposed research aims to evaluate the histopathological effects of the intra-articular administration of recombinant human proteoglycan 4 (rhPRG4) on an experimental model of induced temporomandibular joint (TMJ) osteoarthritis (OA) in rabbits. An experimental study was conducted on rabbit TMJs. Twenty-four TMJs were included, distributed as follows: (1) TMJ-C, a control group consisting of four healthy TMJs; (2) TMJ-OA, a group consisting of five TMJs with OA; (3) TMJ-OA-WT, a group consisting of five untreated TMJ-OAs; (4) rhPRG4-30, a group consisting of five TMJ-OAs treated with rhPRG4 30 μg/mL; and (5) rhPRG4-100, a group consisting of five TMJ-OAs treated with rhPRG4 100 μg/mL. A histopathological analysis was performed, considering the mandibular condyle, articular disc, and mandibular fossa, comparing the groups. In addition, a quantitative comparative analysis was performed using the Osteoarthritis Research Society International (OARSI) scale. Kruskal–Wallis and Dunn’s post hoc tests were used for statistical analysis, considering a statistical significance of *p* < 0.05. The histological analysis of TMJ tissue reveals key differences between healthy, osteoarthritic, and rhPRG4-treated joints. The intra-articular infiltration of rhPRG4 in TMJ-OA has a cartilage and articular disc repair effect, reducing the severity of osteoarthritis and promoting a more organized cartilage structure, with slightly better results at the 30 μg/mL concentration.

## 1. Introduction

The therapeutic use of regenerative medicine or tissue engineering has been increasing in pathologies such as osteoarthritis (OA) of the temporomandibular joint (TMJ). Tissue engineering is based on replacing, manufacturing, or regenerating human cells, tissues, or organs to restore or establish their normal function [1,2]. In its beginnings, regenerative medicine in TMJ-OA was based on implanting tissues into the joint to promote its repair. However, it was necessary to perform joint surgery to introduce the material. Over time, the technique evolved towards infiltrating liquid or semiliquid substances of different viscosities, thus avoiding surgery [1].

Currently, the therapeutic use of regenerative medicine in TMJ-OA is mainly represented by the infiltration of hyaluronic acid (HA), platelet-rich plasma (PRP) or growth factors (GF), and stem cell-based therapies. Of these alternatives, HA and PRP present more significant evidence and are used in daily practice, while the evidence for stem cell therapy is still developing [3,4,5,6,7].

HA is a key lubricant component of synovial fluid in the TMJ. When used as a treatment, it presents essential evidence of its anti-arthritic effect on joint tissues [3,6,8]. Another lubricating molecule that may have reparative or regenerative effects within the components of synovial fluid in TMJ-OA is proteoglycan 4 (PRG4), also known as lubricin.

PRG4 is a high molecular weight mucinous glycoprotein produced by B-type synoviocytes and chondrocytes of the superficial zone of articular cartilage. This protein is found in the synovial fluid (SF) of synovial joints [9] and plays a crucial role in joint lubrication, synovial homeostasis, immunomodulation, and the suppression of inflammation [10,11,12,13]. PRG4 contains core 1 O glycosylations that provide its lubricating function, enabling it to perform multiple essential functions in joints [14]. In addition, it protects cartilage by preventing the deposition of abrasive proteins and facilitates SF energy absorption and dissipation, thus protecting cartilage from mechanical damage [15]. It has also demonstrated anti-proliferative effects, preventing synovial hyperplasia, although the specific mechanisms are not yet fully understood [16].

Recent studies have suggested that PRG4 deficiency is associated with cartilage destruction, and a decrease in PRG4 may be associated with the development of OA. This ability of PRG4 to reduce friction and protect cartilage indicates that it could be a promising therapeutic option for this pathology, providing adequate lubrication and long-term protection against joint degeneration [10,17].

The intra-articular administration of PRG4 has shown promising results in animal models of OA. Different ways of obtaining PRG4 have been described, some related to lubricin biosynthesis via synoviocytes, purified human lubricin, or mostly to full-length recombinant human PRG4 (rhPRG4). Early evidence was observed in rat knees, where intra-articular injection of PRG4 showed significant improvement in reducing joint friction and inhibiting cartilage degeneration [10,13,18,19]. Also, rhPRG4 has been shown to have anti-inflammatory effects in synovial joints. One of these pathways is associated with the inhibition of fibroblast-like synoviocyte proliferation [20,21] or the ability to bind and antagonize Toll-like receptors, decreasing the activation of nuclear factor kappa B (NF-κB) and inflammatory cytokines [22].

On the other hand, a correlation between increased plasma inflammatory cytokines, such as Interleukin-1 beta (IL-1β) and Tumor Necrosis Factor alpha (TNF-α), and decreased plasma PRG4 has been demonstrated [23]. Elevated levels of inflammatory cytokines could trigger molecular processes that decrease PRG4 expression, thus favoring the development of OA. An IL-1 receptor antagonist somewhat restores PRG4 expression in articular cartilage, providing evidence for a correlation between proinflammatory cytokines and PRG4 expression [24].

In preclinical animal models of joint inflammation, a decrease in PRG4 concentration in the SF after joint injury is observed, which is associated with increased damage to the cartilage surface [25]. In patients with acute knee injuries, PRG4 levels in the SF decrease and return to homeostatic levels generally within one year after injury [17].

Regarding the TMJ, PRG4 expression has been identified in healthy and OA articular cartilage. In healthy cartilage, PRG4 is mainly expressed in the superficial zone of the cartilage and, to a lesser extent, in the medial zone. In cartilage with TMJ-OA, no expression was observed in the medial zone, and expression in the superficial zone was decreased [26,27,28,29]. In addition, in PRG4-/- rats, changes in the TMJ tissue were seen from 2 months of development, whereas at 6 months, osteoarthritic degradation is evident in the joint tissue [30].

Despite advances in understanding OA and the therapeutic potential of PRG4, significant work remains to advance this potential in the clinic. Specifically, no studies evaluate the effects of PRG4 administration in TMJ-OA, a condition with unique pathophysiological characteristics due to the complexity of its structure and function. The proposed research aims to evaluate the histopathological effects of the intra-articular administration of rhPRG4 on articular cartilage and articular disc in an experimental model of induced TMJ-OA in rabbits.

## 2. Results

### 2.1. Descriptive Histological Analysis of Articular Cartilage in the Rabbit Temporomandibular Joint Reveals Key Differences Between Healthy and Osteoarthritic Joints

**Figure 1:**
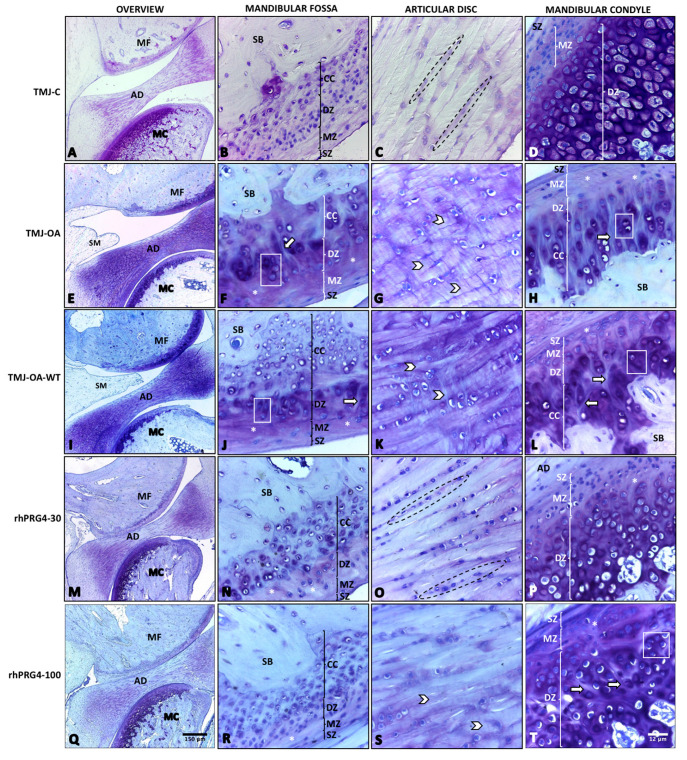
Temporomandibular joint of rabbit (*Oryctolagus cuniculus*). TMJ-C: healthy temporomandibular joints as a control group (**A**–**D**); TMJ-OA: temporomandibular joint with osteoarthritis (**E**–**H**); TMJ-OA-WT: osteoarthritic temporomandibular joint without treatment and evaluated at 30 days after treatment period (**I**–**L**); rhPRG4-30: TMJ-OA joint treated with rhPRG4 30 μg/mL and assessed 30 days after the treatment (**M**–**P**); rhPRG4-100: TMJ-OA treated with rhPRG4 100 μg/mL and evaluated 30 days after treatment (**Q**–**T**); MC: mandibular condyle; AD: articular disc; M: mandibular fossa; SZ: superficial zone; MZ: middle zone; DZ: deep zone; CC: calcified cartilage; SB: subchondral bone; dotted line area: chondrocytes arranged in clusters parallel to the collagen fibers; grid area: chondron clustering near deep fibrillation; asterisk: heterogeneous matrix texture; arrowhead: disorganized collagen fibers; arrow: deep fibrillation. Toluidine Blue stain.

In the control group (TMJ-C), the cartilage was smooth, continuous, and had three well-demarcated zones: the superficial zone (SZ), the middle zone (MZ), and the deep zone (DZ), the latter being the thickest. In contrast, in the TMJ-OA group, a focal discontinuity on the surface and a generalized irregularity was observed, in addition to a thinning of the cartilage and a more fibrous matrix compared to the TMJ-C group. Deep fibrillations and proteoglycan depletion in the matrix were hallmarks of the TMJ-OA group, with hypertrophic chondrocytes and disorganization of collagen fibers. These structural differences suggest significant alterations associated with osteoarthritis in the TMJ-OA group (Table 1), (Figure 1A–D).

### 2.2. Descriptive Histological Analysis of the Rabbit Osteoarthritic Temporomandibular Joint Suggests Beneficial Treatment Effects by Intra-Articular Administration of rhPRG4

Comparing the rhPRG4-30 and rhPRG4-100 groups with the TMJ-OA and TMJ-OA-WT groups, key differences in the condition of articular cartilage and synovial membrane in the TMJ are highlighted. In the TMJ-OA groups, the discontinuity of the cartilage surface, thinning, a more fibrous matrix with deep fibrillations, and signs of proteoglycan depletion, chondrocyte loss, and disorientation were observed (Figure 1E–H). The TMJ-OA-WT group showed greater disease progression with deeper and denser fibrillations (Figure 1I–L). Both groups exhibited synovial membrane thickening due to the increased presence of inflammatory cells and hyperplasia of synovial cells with an increased size.

In contrast, in the rhPRG4-30 and rhPRG4-100 treated groups, repair processes were observed in the articular cartilage, manifesting in a more organized appearance with increased thickness, more uniform surface, and increased cationic staining of the matrix, along with less alteration in the synovial membrane compared to the TMJ-OA and TMJ-OA-WT groups. In the rhPRG4-30 group, some abrasion areas were observed in the SZ. In the MZ of the mandibular condyle, the proliferation of chondrocytes arranged in an isolated manner was observed, suggesting an ongoing repair process. In addition, traces of deep fibrillations were seen in the DZ, indicating an attempt at cartilage tissue regeneration (Figure 1M–P). The articular cartilage of the rhPRG4-100 group also exhibited a repair process; however, increased collagen condensation and fibrillation were noted in deeper areas (Figure 1Q–T).

Both groups demonstrated a potential reparative effect with rhPRG4 treatment in the TMJ affected by OA. However, a non-significant trend toward improved matrix organization and regeneration was observed with the 30 µg/mL rhPRG4 dose (Table 1).

### 2.3. Intra-Articular Administration of rhPRG4 as a Treatment Reduces the Severity of Osteoarthritis in the Articular Cartilage of the Rabbit Temporomandibular Joint

**Figure 2:**
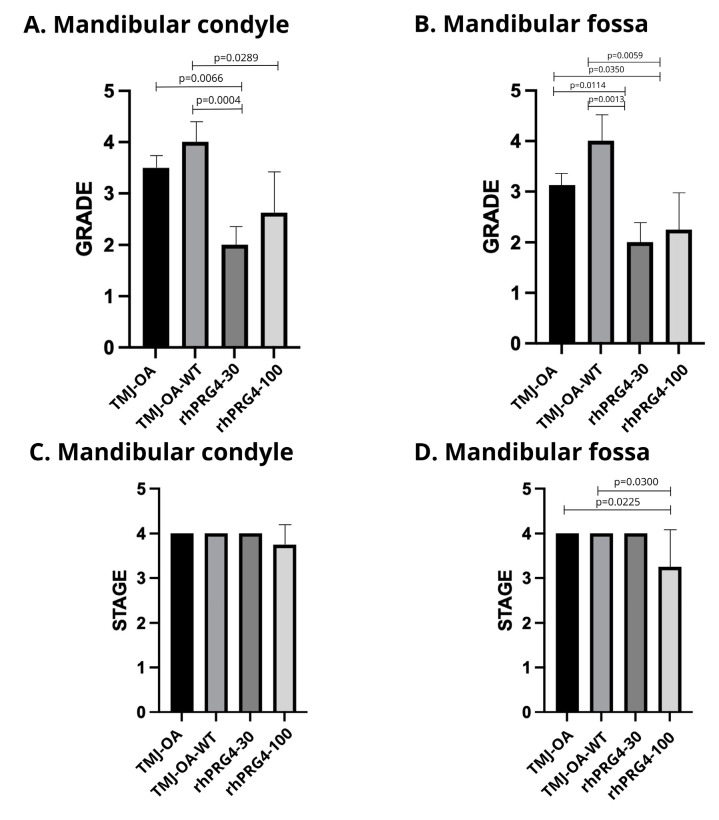
OARSI scale score to evaluate the histopathology of osteoarthritic cartilage in the condyle and mandibular fossa of the temporomandibular joint of the rabbit (*Oryctolagus cuniculus*). (**A**) grade scores of the mandibular condyle; (**B**) grade scores of the mandibular fossa; (**C**) stage scores of the mandibular condyle; (**D**) stage scores of the mandibular fossa; TMJ-OA: temporomandibular joint with osteoarthritis; TMJ-OA-WT: osteoarthritic temporomandibular joint without treatment and evaluated at 30 days after treatment period; rhPRG4-30: TMJ-OA joint treated with rhPRG4 30 μg/mL and assessed 30 days after the treatment; rhPRG4-100: TMJ-OA treated with rhPRG4 100 μg/mL and evaluated 30 days after treatment. GraphPad Prism version 5.00. Significant statistical differences *p* < 0.05.

The OARSI score of articular cartilage in TMJs revealed that both MC and MF exhibited the highest degree of OA in the TMJ-OA and TMJ-OA-WT groups (Figure 2). Specifically, the rhPRG4-30 and PRG4 -100 groups showed a significant reduction in the severity of OA in the articular cartilage of the MC and MF compared to the TMJ-OA and TMJ-OA-WT groups (Figure 2A,B). The TMJ-OA group had no significant difference in OA severity compared to the TMJ-OA-WT group in all structures analyzed (Figure 2). Likewise, the rhPRG4-30 and rhPRG4-100 groups also showed no significant differences in OA severity in both MC and MF (Figure 2A,B). Finally, significant differences in OA stage were only observed between the untreated and rhPRG4-100 groups (Figure 2D).

These results suggest that the intra-articular infiltration of rhPRG4 in osteoarthritic TMJs beneficially affects articular cartilage by reducing the severity of osteoarthritis and promoting a more organized cartilage structure.

## 3. Discussion

The present study is the first to investigate the effects of intra-articular infiltration of rhPRG4 on induced TMJ-OA in rabbits. The results obtained are promising and suggest that rhPRG4 may significantly reduce the severity of OA.

Histological analysis revealed significant improvements in the articular cartilage structure of rabbits with TMJ-OA treated with rhPRG4 compared to untreated groups. The rhPRG4-30 and rhPRG4-100 groups showed a more uniform cartilage surface, a more organized matrix, and increased cartilage thickness. These improvements are consistent with previous studies in the knee that have demonstrated beneficial effects of rhPRG4 in animal models of OA [10,18]. The articular disc also showed significant improvements in the rhPRG4-treated groups. In the untreated groups, the articular disc showed signs of degeneration, such as thinning, matrix disorganization, and proteoglycan loss, consistent with the TMJ-OA literature [31]. In contrast, the rhPRG4-treated groups showed a more uniform and thicker disc structure, suggesting a reparative effect of rhPRG4.

The observed efficacy of rhPRG4 in improving TMJ articular cartilage structure and protecting the articular disc is attributed to its multiple biological functions. rhPRG4 plays a crucial role in lubricating articular surfaces, significantly reducing friction, which protects cartilage from mechanical wear and tear [32]. Furthermore, the ability of rhPRG4 to prevent chondrocyte apoptosis, as Waller et al. demonstrated, is essential for maintaining cell viability in OA-damaged cartilage [33]. rhPRG4 also possesses immunomodulatory properties that can reduce inflammation in the affected joint, a crucial factor in the progression of OA [13]. Chronic inflammation in OA contributes to cartilage degradation and joint dysfunction. By modulating the immune response and reducing inflammation, rhPRG4 protects existing cartilage and promotes a favorable environment for extracellular matrix repair and regeneration.

An interesting observation is comparing rhPRG4 concentrations of 30 μg/mL and 100 μg/mL. Although both groups improved cartilage and articular disc structure, the rhPRG4-30 group showed slightly better regeneration and more coherent matrix organization than the rhPRG4-100 group. This finding suggests that a lower concentration of rhPRG4 may be more effective for cartilage repair, possibly due to a better balance between lubrication and stimulation of matrix synthesis [10,18]. However, further studies are required to find the optimal concentration.

The results of this study show a reduction in the degree of OA according to the OARSI classification and a decrease in the extent of the lesion in a concentration-dependent trend. The OARSI classification evaluates grade (cartilage quality) and stage (extent of damage), allowing us to differentiate between these aspects [34,35]. rhPRG4 appears to be effective in improving cartilage quality, reducing inflammation, and promoting extracellular matrix synthesis [20,21,36]. This targeted action is in line with previous studies [10,18] that have demonstrated the ability of rhPRG4 to improve the structural and functional integrity of existing cartilage.

Currently, treatments for TMJ-OA focus primarily on symptom relief through non-steroidal anti-inflammatory drugs, corticosteroids, and physical therapies, but these approaches do not halt or reverse joint degeneration [37]. PRG4 administration represents a novel therapeutic strategy that could provide benefits beyond simple symptom relief. The results of this study suggest that rhPRG4 not only reduces joint friction and protects cartilage but also promotes repair and regeneration processes in damaged cartilage. This could have a significant impact on improving joint function and reducing long-term pain in patients with TMJ-OA.

Although the results are promising, this study has some limitations that should be considered. Firstly, the experimental model was based on rabbits, and although these animals are suitable for preclinical studies, the results cannot be directly extrapolated to humans. As background, rhPRG4 has been used clinically to manage dry eye disease in humans. Therefore, the potential for rapid translational uptake is significant. It is also important to acknowledge that the absence of a vehicle-only control group represents a limitation of this study. This omission prevents us from fully isolating the specific effects of rhPRG4 from potential non-specific influences related to the intra-articular injection procedure, such as the volume of the vehicle, joint manipulation, or mechanical stimulation. While the study design sought to minimize the number of animals in accordance with ethical guidelines, future studies including a vehicle control group will be essential to confirm the specificity and reproducibility of rhPRG4’s therapeutic effects. In addition, the study focused on a relatively short period of treatment and follow-up, so long-term studies are required to assess the sustainability of the beneficial effects of PRG4. Further studies using advanced imaging techniques and molecular analysis are also needed to deepen the understanding of the mechanisms behind the efficacy of rhPRG4. These techniques may provide more detailed insight into how PRG4 affects cartilage architecture and cellular dynamics at the microstructural level. In addition, research exploring combinations of PRG4 with other therapeutic agents, such as hyaluronic acid or growth factors, could offer synergistic approaches to maximize joint repair and prevent OA progression.

## 4. Materials and Methods

### 4.1. Animals

An experimental study was conducted on 12 healthy male *Oryctolagus cuniculus* rabbits weighing approximately 3 kg and eight months of age, according to the recommendations described by Poole et al. [38]. The animals were kept in a controlled environment regarding temperature, environmental noise, and a 12 h light/12 h dark cycle. The animals were housed in cages individually and randomly assigned to each group. Simple random sampling was used to allocate the animals to the experimental groups; each animal was assigned a number. All animals used in this study were male in order to minimize biological variability associated with hormonal fluctuations in females, which may influence the progression and severity of osteoarthritis. Sex-related differences, particularly due to hormonal and biomechanical factors, can significantly impact OA phenotypes [39]. Thus, the use of male rabbits aimed to reduce potential confounding variables and ensure more consistent and interpretable results.

The animals were under the care of a veterinarian, and the guidelines of Animal Research: Reporting of In Vivo Experiments (ARRIVE) guidelines and the National Research Council Guide for the Care and Use of Laboratory Animals [40] were followed. The study was performed at the experimental surgery unit of the Center of Excellence in Morphological and Surgical Studies of the Universidad de La Frontera, Chile, with the approval of the Scientific Ethics Committee of the Universidad de La Frontera (File N° 027_21) on 7 April 2021.

**Figure 3:**
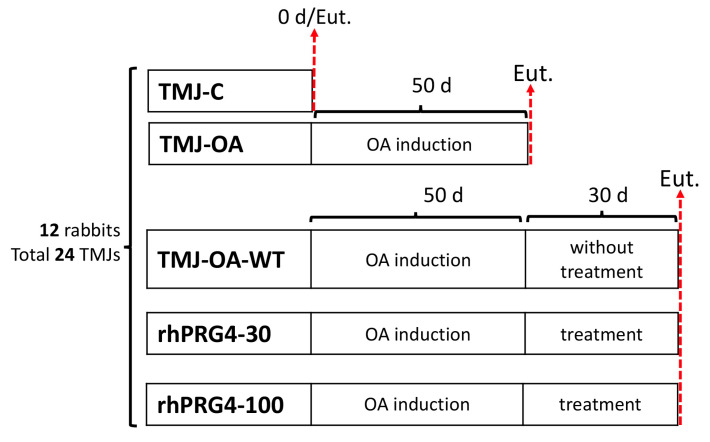
Experimental design and grouping of temporomandibular joints (TMJs) in rabbits. Twelve rabbits (24 TMJs) were divided into five groups. TMJ-C: healthy temporomandibular joints as a control group; TMJ-OA: osteoarthritic temporo-mandibular joint; TMJ-OA-WT: osteoarthritic temporomandibular joint untreated and evaluated 30 days after treatment period; rhPRG4-30: osteoarthritic temporomandibular joint treated with rhPRG4 30 μg/mL and assessed 30 days after treatment; rhPRG4-100: osteoarthritic temporo-mandibular joint treated with rhPRG4 100 μg/mL and evaluated 30 days after treatment. d: days; Eut.: euthanasia.

The two TMJs of each animal were considered to reduce the sample size and meet the experimental criteria established by Russell and Burch [41]. A sample size calculation was performed considering up to 10% losses and a statistical significance of *p* < 0.05. According to the above, twenty-four TMJs were included, distributed as follows: (1) TMJ-C, a control group consisting of four healthy TMJs; (2) TMJ-OA, a group consisting of five TMJs with OA; (3) TMJ-OA-WT, a group consisting of five untreated TMJ-OAs; (4) rhPRG4-30, a group consisting of five TMJ-OAs treated with rhPRG4 30 μg/mL; and (5) rhPRG4-100, a group consisting of five TMJ-OAs treated with rhPRG4 100 μg/mL. The distribution of the groups is shown in Figure 3. Protocols for TMJ-OA induction, intra-articular rhPRG4 administration, histological processing, and histological analysis were performed according to previously described protocols [3,4,35,42,43].

### 4.2. TMJ-OA Induction

Except for the TMJ-C group, TMJ-OA induction was performed in all groups. Animals were anesthetized intramuscularly with ketamine (40 mg/kg), xylazine (5 mg/kg), and acepromazine (1 mg/kg), and the TMJ area was shaved and disinfected with 70% ethyl alcohol. At that time, 50 µL of sodium mono-iodoacetate (MIA) at a concentration of 3 mg/mL was infiltrated into the joint space with a 22-gauge needle. A period of 50 days was allowed to elapse to develop TMJ-OA [42,44]. After a 50-day waiting period, the animals in the TMJ-OA group were euthanized and subsequently analyzed. In the TMJ-OA-WT, rhPRG4-30, and rhPRG4-100 groups, after the initial 50-day period, an additional 30 days were taken before euthanasia and analysis (Figure 3).

### 4.3. Intra-Articular rhPRG4 Administration

Intra-articular rhPRG4 administration was conducted using the same protocol for the anesthesia and previously mentioned preoperative measures. The infiltration technique was standardized, considering the caudal margin of the orbital lamina as the anatomical reference point. It was directed 5 mm caudally and 1 mm ventrally, with the needle at a 45° angle towards the ventral concerning the skin [9]. In the rhPRG4-30 group, 0.1 mL of rhPRG4 at 30 μg/mL (Lµbris BioPharma^®^, Framingham, MA, USA) was injected into the joint space with a 22-gauge needle. Similarly, in the rhPRG4-100 group, 0.1 mL of rhPRG4 at 100 μg/mL (Lµbris BioPharma^®^, Framingham, MA, USA) was injected.

### 4.4. Histological Processing

Once the experimental protocol was completed, the animals were sacrificed, and the joint tissue was dissected. No animal losses, adverse effects, or modifications were reported. Samples were coded to maintain process masking during evaluation. The tissue was fixed with 10% buffered formalin (1.27 mol/L formaldehyde in 0.1 M phosphate buffer, pH 7.2) for 48 h. TMJs were then decalcified in 10% ethylenediaminetetraacetic acid (EDTA) (in 0.1 M phosphate buffer 7–8) in ultrasonic decalcification (Use 33, Medite, Burgdorf, Germany) for 30 days. Subsequently, samples were dehydrated in an ascending alcohol battery, rinsed in xylene, and embedded in Paraplast Plus (Sigma-Aldrich Co., St. Louis, MO, USA). Serial sections of the TMJs were cut in the parasagittal plane at a thickness of 5 μm using a microtome (Leica^®^ RM 2255, Leica Biosystems, Deer Park, IL, USA). To optimally grade the OA, successive sections of the deeper planes of the joint were stained and visualized under a light microscope. Then, one section per joint was selected for the more detailed analysis, considering the plane of the block that crosses the lesion to the greatest extent and shows the most pronounced alterations [34]. To reduce selection bias and ensure that the analyzed section was representative of the osteoarthritic changes, the section chosen was the one that best captured the full extent of the lesion. This approach, based on the OARSI histopathological assessment system, provides a standardized and reproducible method to evaluate the most affected region of the joint. After selection, the sections were processed and stained with Toluidine Blue for histological evaluation. An optical microscope (Leica^®^ DM 2000 LED, Wetzlar, Germany) was used for visualization, and the slides were photographed with a digital camera (Leica^®^ MC 170 HD, Wetzlar, Germany). To maintain blinding in the analysis, the slides were also coded.

### 4.5. Histological Analysis

Histological slides were randomly analyzed for each TMJ to compare the different groups. A descriptive analysis of the mandibular condyle (MC), articular disc (AD), and mandibular fossa (MF) was performed. The cartilage was described from superficial to deep, beginning with the superficial zone (SZ), also known as the tangential zone, followed by the mid-zone (MZ), also called the transitional zone; the deep zone or radial zone (DZ); calcified cartilage; and subchondral trabecular bone. Regarding AD, the central zone (CZ) at its thinnest point and the anterior and posterior peripheral zone (PZ) areas were analyzed.

Authors V.I. and B.V. independently and blindly performed the quantitative histological analysis of each sample. In the case of disagreements, these were discussed until reaching a consensus. The interobserver calibration was performed in three phases before analyzing the samples: theoretical training, laboratory training, and calibration (final inter-agreement kappa 0.865).

The OARSI scale was used to grade the articular cartilage grade and stage for both MC and MF. This scale classifies the different cartilage states, where grade 0 corresponds to normal tissue and grades 1 to 6 are relative to OA. Grades 1 to 4 of OA involve changes in cartilage only, while grades 5 and 6 also include the subchondral bone. The OA stratification method, also defined by the OARSI scale, categorizes the disease into four stages based on the horizontal extent of the affected cartilage surface, regardless of the degree of underlying OA. Stage 1 represents less than 10% involvement; stage 2 represents 10 < 25% involvement; stage 3 represents 25–50% involvement; and stage 4 represents greater than 50% involvement. MC and MF cartilage were evaluated separately for each slide in each group [34,35].

### 4.6. Statistical Analysis

Quantitative analysis was performed by calculating the median and interquartile range of the scores obtained on the OARSI scale. Nonparametric inferential statistics were used to compare the degree and stage of TMJ-OA between groups using the Kruskal–Wallis and Dunn’s post hoc tests. The analysis was performed with the STATA 18 program, considering a significance level of α = 0.05. 

## 5. Conclusions

The findings of this preclinical study demonstrate that the intra-articular administration of rhPRG4 has a significant therapeutic effect on TMJ-OA induced in rabbits. Compared to untreated groups, animals that received rhPRG4 showed a reduction in the severity of osteoarthritis, evidenced by the improved structural organization of the articular cartilage, increased cartilage thickness, and regeneration of the articular disc. These reparative effects were more pronounced in the group treated with the 30 μg/mL concentration, suggesting that lower doses of rhPRG4 may provide a more favorable balance between the lubrication and stimulation of matrix synthesis.

The results support the potential of rhPRG4 as an innovative and effective strategy for the treatment of TMJ-OA, beyond conventional symptomatic management. However, further clinical studies are needed to validate its safety, efficacy, and optimal dosing in humans.

## Figures and Tables

**Table 1:** Histological characteristics of the temporomandibular joint tissue according to the groups under study.

Structure		TMJ-C	TMJ-OA	TMJ-OA-WT	rhPRG4-30	rhPRG4-100
Mandibular condyle	SZ	Smooth and continuous surface. Flattened surface cells and a thin layer of fibrous connective tissue.	Abrasion, matrix focal discontinuity. Fibrillations towards the deep zone.	Abrasion, matrix focal discontinuity. Fibrillations towards the deep zone covering the entire articular cartilage.	Slightly irregular surface. Abrasion of the surface layer in some areas. Small and flat or round chondrocytes, aligned parallel to the collagen fibers and the surface.	Slightly irregular surface. In some sectors it is possible to observe abrasion. Small and flat or round cells. In some samples it is observed, focally, areas of collagen condensation whose fibers are directed to the middle and/or deep zone.
	MZ	Undifferentiated cells and spherical chondrocytes in a proteoglycan matrix.	Reduced cellularity with deep fibrillations.	Reduced cellularity, more deep and higher density fibrillations.	Proliferation of chondrocytes, arranged in isolation.	Anisocytosis and proliferation of undifferentiated cells. Clusters of chondrocytes are seen in some samples. Matrix rarefaction, with areas of increased cationic staining around the chondrons. Condensation of collagen fibers.
	DZ	It presents round and larger chondrocytes, organized in isogenic groups. Deep hypertrophic chondrocytes are observed.	Less cellularity. Hypertrophic chondrocytes, forming clusters. Rarefaction and condensation of collagen fibers.	Less cellularity and chondrocyte clusters. Rarefaction and condensation of collagen fibers.	Vestiges of deep fibrillations. Focal rarefaction, increased collagen formation and cationic staining around the chondrons. Some hypertrophic chondrocytes.	Loss of orientation of the chondrons in a disorganized matrix. Increased density of chondrocytes, with decreased cell size. Vestiges of deep fibrillations.
Articular disc	CZ	Chondrocyte stacking and arrangement in parallel (rows) as to collagen fibers.	Focal edema. Increased number and density of collagen fibers. Disorganized fibers. Less cellularity, with randomly arranged hypertrophic chondrocytes.	Focal edema. Increased number and density of collagen fibers. Disorganized fibers. Less cellularity, with randomly arranged hypertrophic chondrocytes.	Collagen fibers arranged in parallel with chondrocytes aligned to them. Chondrocytes are found within cartilage matrix.	Disorganization of collagen fibers and edema. Less cellularity, with some chondrocytes arranged randomly and others parallel to the collagen fibers.
	PZ	More abundant and dense chondrocyte rows.	Randomly arranged hypertrophic chondrocytes within disorganized collagen fibers. Presence of connective tissue with abundant fibroblasts.	Less peripheral connective tissue. Hypertrophy of the synovial membrane.	Randomly arranged hypertrophic chondrocytes within disorganized collagen fibers.	Some hypertrophic chondrocytes randomly arranged among disorganized collagen fibers.
Mandibular fossa	SZ	Fibrous connective tissue, collagen fibers parallel to the surface with intermingled fibrocytes.	Thickness, fiber disorganization, and edema. Rarefaction. Fibrillations that reach the deep zone of the cartilage.	Thickness, fiber disorganization and rarefaction. Fibrillations that reach the deep zone of the cartilage.	Slightly irregular surface. The limits between SZ and MZ are not very evident. Hypocellularity.	Slightly irregular surface. Unclear boundaries between SZ and MZ. Scarce cellularity with anisocytosis.
	MZ	Undifferentiated cells.	Diffuse, with reduced thickness and scarce cellularity.	Diffuse, reduced thickness, and scarce cellularity.	Less thickness and hypocellularity, with rarefaction and edema.	Heterogeneous matrix, with focal edema, low cellularity, and anisocytosis.
	DZ	Chondrocytes immersed in matrix rich in collagen fibers.	Clusters of hypertrophic chondrocytes. Rarefaction and condensation of collagen fibers. Presence of deep fibrillations.	Deep fibrillations. rarefaction and collagen condensation. Clusters of hypertrophic chondrocytes.	Osteoarthritic features are observed, similar to MZ.	Heterogeneous matrix, traces of focal fibrillations. Small chondrocytes distributed mainly in isolation.

TMJ-C: healthy temporomandibular joints as a control group; TMJ-OA: osteoarthritic temporomandibular joint; TMJ-OA-WT: osteoarthritic temporomandibular joint untreated and evaluated 30 days after treatment period; rhPRG4-30: osteoarthritic temporomandibular joint treated with rhPRG4 30 μg/mL and assessed 30 days after treatment; rhPRG4-100: osteoarthritic temporomandibular joint treated with rhPRG4 100 μg/mL and evaluated 30 days after treatment; SZ: superficial zone; MZ: mid zone; DZ: deep zone; CZ: central zone; PZ: peripheral zone.

## Data Availability

The original contributions presented in this study are included in the article. Further inquiries can be directed to the corresponding authors.

## Abbreviations

The following abbreviations are used in this manuscript:

OA	Osteoarthritis
TMJ	Temporomandibular joint
TMJ-OA	Temporomandibular joint osteoarthritis
PRG4	Proteoglycan 4
rhPRG4	Recombinant human PRG4
rhPRG4-30	rhPRG4 30 μg/mL
rhPRG4-100	rhPRG4 100 μg/mL
SF	Synovial fluid
HA	Hyaluronic acid
PRP	Platelet-rich plasma
GP	Growth factors
NF-κB	Nuclear factor kappa B
IL-1β	Interleukin-1 beta
TNF-α	Tumor Necrosis Factor alpha
MC	Mandibular condyle
MF	Mandibular fossa
SZ	Superficial zone
MZ	Middle zone
DZ	Deep zone
AD	Articular disc
CZ	Central zone
PZ	Peripheral zone
OARSI	Osteoarthritis Research Society International
ARRIVE	Animal Research: Reporting of In Vivo Experiments
EDTA	Ethylenediaminetetraacetic acid
